# Front Is High and Back Is Low: Sound-Space Iconicity in Finnish

**DOI:** 10.1177/00238309231214176

**Published:** 2023-12-06

**Authors:** Lari Vainio, Markku Kilpeläinen, Alexandra Wikström, Martti Vainio

**Affiliations:** 1 Perception, Action & Cognition Research Group, Department of Psychology and Logopedics, Faculty of Medicine, University of Helsinki, Finland; 2 Phonetics and Speech Synthesis Research Group, Department of Digital Humanities, Faculty of Arts, University of Helsinki, Finland

**Keywords:** Spatial language, sound symbolism, speech, vowel production

## Abstract

Previous investigations have shown various interactions between spatial concepts and speech sounds. For instance, the front-high vowel [i] is associated with the concept of forward, and the back-high vowel [o] is associated with the concept of backward. Three experiments investigated whether the concepts of forward/front and backward/back are associated with high- and low-pitched vocalizations, respectively, in Finnish. In Experiments 1 and 2, the participants associated the high-pitched vocalization with the forward-directed movement and the low-pitched vocalizations with the backward-directed movement. In Experiment 3, the same effect was observed in relation to the concepts of front of and back of. We propose that these observations present a novel sound-space symbolism phenomenon in which spatial concepts of forward/front and backward/back are iconically associated with high- and low-pitched speech sounds. This observation is discussed in relation to the grounding of semantic knowledge of these spatial concepts in the movements of articulators such as relative front/back-directed movements of the tongue.

## 1 Introduction

When a child is swinging, sometimes either a child or a person who is pushing the swing to increase the swinging speed is vocalizing, in a looped sequence, the high-pitched /ki:/ and low-pitched /kɑ:/ one after another in the rhythm of swinging. At this point, it should be emphasized that in this study, these kinds of high- and low-pitched vocalizations refer to relative heightening or lowering of F0 (fundamental frequency) when vocalizing with related changes in other acoustic aspects, such as intensity, first formant frequency (F1), and second formant frequency (F2). This phenomenon can be associated at least with Finnish culture. Based on anecdotal evidence (i.e., personal observation), it seems possible that the high-pitched /ki:/ is typically vocalized when the swing moves forward and the low-pitched /kɑ:/ is vocalized when the swing moves backward. This study investigates whether this assumption can be supported by empirical evidence. More precisely, the study explores whether high-pitched vocalizations are associated with forward-directed movement and low-pitched vocalizations are associated with backward-directed movement, and if these associations are observed, what might be the underlying reason for them. Furthermore, the study investigates whether this potential phenomenon could be observed in relation to the concepts of front/back. The concepts of front and back are semantic counterparts for the concepts of forward and backward, respectively. The main difference between them is that forward/backward refers to directional movement knowledge, whereas front/back refers to a fixed spatial position of a target object in relation to a reference object.

Some might ask why it is important to know whether a high-pitched vocalization is associated with forward/front and a low-pitched vocalization is associated with backward/back. One reason is that this phenomenon might be taken to present an unexplored case of language iconicity. In linguistics, iconicity refers to the conceived similarity between the form of a linguistic sign and its meaning. Sign language (Perniss et al., 2010) and co-speech gestures (McNeill, 2008) include a wide variety of body movements in which the movement iconically represents the referent. However, spoken language can correspondingly contain speech sounds that iconically represent the referent. The phenomena that reveal a systematic association between a speech sound and meaning are often referred to as sound symbolism. Onomatopoeic words (e.g., knock and meow) provide the most obvious demonstration of these kinds of speech sounds. Furthermore, sound-meaning iconicity can be classified as absolute (i.e., a direct relation between meaning and speech sound as in onomatopoeic words) and relative (i.e., related speech sounds are associated with related meanings as in the sound-meaning mappings of the sound-size symbolism that is introduced below; Gasser et al., 2005; Lockwood & Dingemanse, 2015). Perhaps the most famous example of this kind of relative iconicity of speech sounds—the sound-size symbolism—associates, small magnitudes with front-high vowels and large magnitudes with low and back vowels (Knoeferle et al., 2017; E. Sapir, 1929). There is nothing obviously small in front-high vowels, except the relatively small oral cavity (Kawahara, 2021) as discussed below, yet plenty of studies show that these speech sounds are systematically linked to small magnitudes. Finally, in addition to iconicity, the non-arbitrary association between speech sounds and meaning can be based on systematicity in which statistical regularities in the patterns of speech sound predict function (e.g., prosodic cues that help to distinguish nouns from verbs; Dingemanse et al., 2015). However, it is important to clarify that this paper solely focuses on the relative iconicity of language.

The underlying mechanisms behind the sound-size symbolism and other sound symbolism effects that reflect the relative iconicity of language are, however, still largely under debate (see Sidhu & Pexman, 2018 for a review). As an example, some researchers suggest that the sound-size symbolism is based on acoustic features of vocalization. According to this view, low-back vowels are associated with larger concepts than high-front vowels because the intrinsic pitch of low-back vowels is typically lower than the intrinsic pitch of high-front vowels (Whalen & Levitt, 1995) making them a more suitable linguistic match to large concepts (notice that large things and animals typically produce lower-pitched sounds than small things and animals; Ohala, 1994; Pisanski et al., 2014). In contrast, the articulatory explanation of the sound-size symbolism assumes that low-back vowels are associated with larger concepts than high-front vowels because the manner of articulation, in relation to the size of the oral cavity, makes them a more suitable match to large concepts (Ramachandran & Hubbard, 2001; E. Sapir, 1929). In the context of iconicity, the earlier explanation highlights that the acoustic feature of the vowel associates the vowel iconically with the magnitude, whereas the latter explanation highlights that the articulatory feature of the vowel associates the vowel iconically with the magnitude. In addition, of course, it is possible that both of these mechanisms contribute to the effect.

Correspondingly to the sound-size symbolism, it is possible that high-pitched vocalization can be associated with forward-directed movement and low-pitched vocalization can be associated with backward-directed movement. If this were the case, the linkage between the vocalization pitch and spatial meaning could be similarly based on acoustic and/or articulatory features of the speech sound. For instance, high-pitched vocalization can be associated with the concept of forward because a high pitch iconically refers—for one reason or another—forward-directed movement. Alternatively, it can be associated with the concept of forward because this concept is implicitly associated with some articulatory movements that are involved in producing high-pitched vocalizations. Therefore, in addition to exploring whether high/low-pitched vocalizations are systematically associated with the concepts of forward/front and backward/back, the study also investigates whether the manner of articulation might provide a causal explanation for this potential interaction by analyzing acoustic characteristics of vocalizations that are reflecting movements of articulatory organs.

Experiment 1 uses a two-alternative forced choice task to investigate whether high-pitched vocalization is associated with forward-directed movement and low-pitched vocalization is associated with backward-directed movement, as assumed based on anecdotal evidence. In the task, the participants are aurally presented with vocalization of high-pitched /ki:/ and low-pitched /kɑ:/ one after another in a looped sequence. Simultaneously, the participants are visually presented with two animated videos. In one video, the forward-directed movement of the swinging is presented with the /ki:/ sound of the auditory stimulus, and the backward-directed movement is presented with the /kɑ:/ sound of the auditory stimulus. In the other video, the sound-to-direction mapping is reversed. The earlier mapping condition is hypothesized to present congruence between sound and movement, whereas the latter mapping condition is hypothesized to present incongruence between sound and movement. The participants are required to select the video that intuitively provides a better match to the auditory stimuli. It is hypothesized that if it is indeed the case that high-pitched vocalization is associated with forward-directed swing movement and low-pitched vocalization is associated with backward-directed swing movement, the participants should select the congruent video significantly more often than the incongruent video.

Experiment 2 uses the stimulus–response compatibility method (Kornblum et al., 1990) to explore whether the phenomenon investigated in Experiment 1 can be observed in the context of producing—instead of perceiving—high- and low-pitched vocalizations. Therefore, the participants were presented with stimuli showing a forward or backward-directed movement. They were asked to vocalize either a high- or low-pitched [æ], which is a front-low vowel, based on whether the movement was directed forward or backward. The participants carry out this task in two blocks from which one requires hypothetically congruent responding (i.e., high-pitched vocalization—forward, low-pitched vocalization—backward) and the other requires hypothetically incongruent responding (i.e., low-pitched vocalization—forward, high-pitched vocalization—backward). It is hypothesized that if the high-pitched vocalizations are indeed associated with forward-directed swing movement and the low-pitched vocalizations are associated with backward-directed swing movement, the vocal responses should be produced faster in the congruent conditions.

Finally, Experiment 3 used a similar task to that of Experiment 2 with the exception that the vocal responses were performed based on whether the target object was at the front of or back of the referent object. Hence, this final experiment explores whether the potential interaction between pitch production and the spatial concepts of forward and backward is an exclusive sound-space symbolism phenomenon, which is only linked to spatial concepts that refer to directional movements. Alternatively, it is possible that the interaction is a phenomenon, which is generalizable to the spatial concepts of front and back, which are semantically tightly linked to the concepts of forward and backward.

In addition to analyzing the reaction times of Experiments 2 and 3, we also analyzed the vocal characteristics of intensity (dB), F0, F1 (the first formant), and F2 (the second formant). Regarding F1 and F2, it is known that vocalizations that are produced with the front position of the tongue (e.g., [i]) typically have higher F2 values than vocalizations that are produced with the back position of the tongue (e.g., [o]), whereas F1 values are heightened when vocalizations are produced with an increased oral cavity (e.g., [ɑ]) in comparison with decreased oral cavity (e.g., [i]; Fant, 1960). It could be hypothesized, for example, that if the perceived movement that is directed forward is associated with high-pitched vocalization, F0 values of vocalizations could be higher in relation to vocalizations that are produced to forward-directed movements in comparison to backward-directed movements. In addition, it could be hypothesized, for example, that if the perceived movement that is directed forward is associated with the articulatory front position of the tongue, F2 values of vocalizations could be higher in relation to vocalizations that are produced to forward-directed movements in comparison to backward-directed movements.

## 2 Experiment 1

### 2.1 Method

#### 2.1.1 Participants

Twenty volunteers participated in the experiments (20–41 years of age; mean age = 26 years; three males; three left-handed). All participants had normal or corrected-to-normal vision and hearing and were native Finnish speakers. The selection of the sample size was based on a similar study using a forced-choice matching task to investigate how sound-symbolic features of a listened sound influence selecting one of the two alternative action videos (Imai et al., 2008; Experiment 1b). That study used 15 participants, and a similar number of experimental trials as this study, to show that the action, which is matching to the sound, is selected significantly (*p* < .01, *d* = 0.90) above chance. Based on this, the sample size calculation carried out by using G*Power software (Faul et al., 2007), proposed that already 16 participants would suffice to produce a sound-action effect, which is significantly above chance, using a similar forced-choice matching task. We obtained written informed consent from all participants. The study was approved by the Ethical Review Board in Humanities and Social and Behavioral Sciences at the University of Helsinki.

#### 2.1.2 Stimuli, procedure, and apparatus

**Figure 1.**
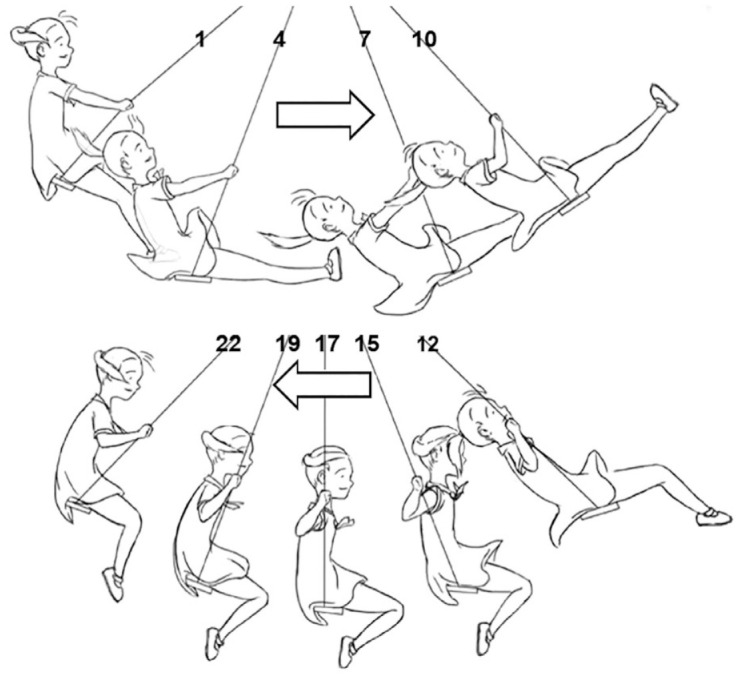
In Experiment 1, a single back-and-forth movement was animated from 22 pictures. *Note*. This figure presents nine of them. The swing movement started from Image 1 or 12. The arrow shows the direction of the swing movement.

The visual stimuli consisted of four different swinging animations. In two of them, the face of the swinging girl was pointing to the left and in two of them, it was pointing to the right. The left- and right-pointing stimuli consisted of two animation stimuli from which one started with picture 1 (i.e., the movement direction was congruent with the sound) and one started with picture 12 (i.e., the movement direction was incongruent with the sound; see Figure 1). The girl swung 18 times back and forth (or forth and back) in each animation. The entire animation lasted for 40.86 s and consequently, the single back-and-forth movement lasted for 2.27 s. The single back-and-forth movement was animated from 22 pictures.

**Figure 2.**
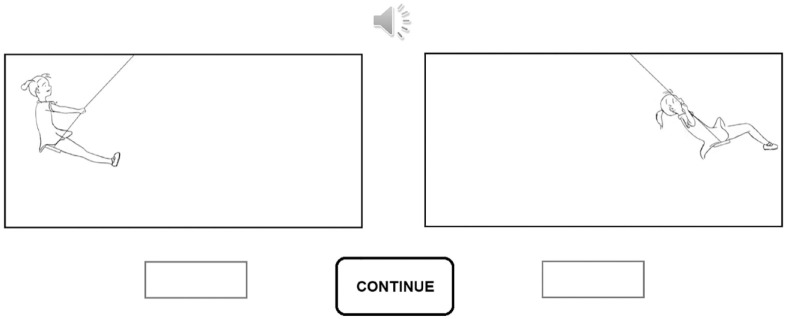
The illustration of one possible trial of Experiment 1. *Note*. The details of the design are described in the Method section of Experiment 1.

The experiment consisted of eight animation pairs. In each pair, one animation was congruent with the sound (i.e., the /ki:/ component of the vocalization was presented with the forward-directed movement) and one was incongruent with the sound. Hence, both congruent animations (i.e., the left- and right-pointing) were presented four times in the experiment. In each trial, one animation was presented on the left side and one was presented on the right side of the display (see Figure 2). The left–right locations of the congruent animation stimuli were pseudorandomized so that the same number of congruent animations was located on the left and right sides of the display. The presentation order of the left- and right-pointing congruent animation stimuli was randomized. The pairing of each congruent animation with an incongruent animation was pseudorandomized so that both incongruent animations (i.e., the left- and right-pointing) were presented four times in the experiment and they were paired with the congruent animations in randomized order.

The auditory stimulus consisted of /ki: kɑ:/ vocalization, in a looped sequence, produced by a male. The /ki:/ component of the vocalization had a higher pitch (*M*: 129 Hz) than the /kɑ:/ component of the vocalization (*M*: 92 Hz). The /ki:/ and /kɑ:/ components had the same duration (960 ms) and intensity (58.3 dB) modified by Praat. The mean F1 was 225 Hz, and the mean F2 was 2,303 Hz for the /ki:/ component, while the mean F1 was 788 Hz, and the mean F_2_ was 1,000 Hz for the /kɑ:/ component. The waveform and spectrogram files of the /ki:/ and /kɑ:/ components are provided in the repository (https://osf.io/u7p85/) for closer inspection. The timing of the auditory stimulus was synchronized with the visual stimuli so that, in the congruent conditions, the /ki:/ component started with the presentation of Picture 1 and the /kɑ:/ component started with the presentation of Picture 12. In the incongruent conditions, the /ki:/ component started with the presentation of Picture 12 and the /kɑ:/ component started with the presentation of Picture 1.

Each participant sat in a dimly lit room with his or her head 75 cm in front of a 25-inch Full HD monitor (screen refresh rate: 240 Hz; screen resolution: 1,920 × 1,080). The auditory stimulus was presented over headphones at ca. 75 dB SPL. The size of the visual stimuli (i.e., the swinging girl) was 2.4° (measured from the swing seat to the top of the head). The auditory stimulus and the visual animations were looped for 40.86 s, or until the participant pressed the “continue” button (see Figure 2). There was a frame presenting a “*Ready to continue?*” text between each frame. The next stimuli were initiated by pressing the continue button. The response was performed by inserting the letter x into the response box that was located under the animation, which was assumed to provide a better match to the sound.

The participants were briefed that they were to be presented with a sound and two animation videos in which a girl is swinging. The participants were asked to intuitively indicate which one of the two animations presents a better match to the sound. They were told that there are no right or wrong answers. They were further informed that they could take as much time as they needed to respond, but that after 40 s the stimuli would come to a halt and after that, they should provide the response at the latest. However, they were also advised that there was no reason for them to delay their response if they had a “gut feeling” about the matching stimuli. In that case, they could respond immediately. At the end of the study, the participants were asked whether they were aware that high-[ki:] and low-[kɑ:] are vocalized during swinging movement, and whether they were aware that high-[ki:] is vocalized when the swing moves forward and low-[kɑ:] is vocalized when the swing moves backward. The data are available in the following repository (https://osf.io/u7p85/).

### 2.2 Results

The data were analyzed using the one-sample Wilcoxon signed rank test because the data were not normally distributed. For analysis purposes, the participants’ responses were converted into percentages of sound-symbolically matching responses. The test revealed that the participants selected the matching action significantly above the chance level (*M* = 88.1%, *SD* = 14.9, *z* = 3.89, *p* < .001). Only one participant did not select the matching actions more frequently than the non-matching actions. Fifty percent of her responses were sound-symbolically matching the sound. As many as 10 participants selected the matching action in every trial. This outcome shows that a high-pitched [i] is associated with forward-directed movement and a low-pitched [ɑ] is associated with backward-directed movement. Finally, all participants were aware that high-[ki:] and low-[kɑ:] are vocalized during swinging movement. These responses might be somewhat primed by the experiment. However, none of the participants were aware that high-[ki:] is vocalized when the swing moves forward and low-[kɑ:] is vocalized when the swing moves backward, although most of them noticed that this movement-sound arrangement is very intuitive.

### 2.3 Discussion

The results of Experiment 1 provided empirical evidence to support the anecdotal assumption that high-pitched vocalizations are associated with the forward-directed swing movement, whereas low-pitched vocalizations are associated with the backward-directed swing movement. It should be, however, emphasized that this finding should be replicated in laboratories outside of Finland to investigate to what extent this finding is a cultural phenomenon, and can be observed universally. It is also noteworthy that in addition to presenting high- and low-pitched components, the auditory stimuli also included a difference in vowel content. The high-pitched vocalization always appeared together with the /ki:/ vocalization and the low-pitched vocalization appeared together with the /kɑ:/ vocalization. Hence, one might argue that the vocalization pitch does not contribute to the observed effect, but rather the effect is based on the vowel content of the auditory stimuli so that the front vowel [i] is associated with the forward-directed movement and the back vowel [ɑ] is associated with the backward-directed movement.

Experiment 2 aims to test whether the effect observed in Experiment 1 can be replicated in the context of vowel production when the vowel content remains the same in relation to the high- and low-pitched vocalizations. In comparison with Experiment 1, Experiment 2 recruits the stimulus–response compatibility paradigm in which participants were required to produce the vowel [æ] with either high or low F0, henceforth referred to as high or low pitch, according to the swing direction. The distinction of high or low pitch means here a distinction between two types of utterances of the target vowel that are audibly contrastive with each other based on differences in pitch height. In one block, these responses are performed in the hypothetically congruent condition (e.g., high pitch—forward-directed movement), and in the other block, the responses are performed in the hypothetically incongruent condition. We assume that responses are performed more rapidly in the congruent condition than in the incongruent condition. In addition, we analyze the vocal characteristics of F0, intensity, F1, and F2 to explore whether these vocal characteristics can be modulated by the swing direction.

## 3 Experiment 2

### 3.1 Method

#### 3.1.1 Participants

Twenty-two volunteers naïve to the purposes of the experiment participated in Experiment 2 (19–41 years of age; mean age = 25.1 years; five males; one left-handed). All participants were native speakers of Finnish and reported normal hearing and normal or corrected-to-normal vision. Power was estimated based on simulations (Brysbaert & Stevens, 2018). The simulations were based on an earlier data set from an experiment with a very similar design (L. Vainio et al., 2023). The simulation script and the data set are provided in the following repository: https://osf.io/u7p85/. In the simulations, a mixed linear model with log-transformed reaction time data was fitted. The participants had a random effect on the intercept and the slope of congruency. The simulations suggest, first, that with the effect size (*d_z_* = 0.42) observed by L. Vainio et al. (2023), 22 observers would have sufficed to produce a statistically significant difference in 85% of experiments. The simulations were run with R package simr (Green & MacLeod, 2016). Written informed consent was obtained from all participants. The study was conducted according to the principles expressed in the Declaration of Helsinki. The study was approved by the Ethical Review Board in the Humanities and Social and Behavioral Sciences at the University of Helsinki.

#### 3.1.2 Stimuli, procedure, and apparatus

**Figure 3.**
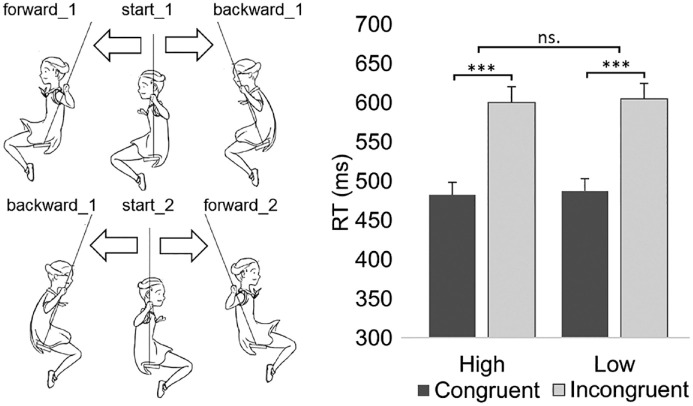
(Left) The stimuli used in Experiment 2. (Right) The mean vocal reaction times for Experiment 2 as a function of the response (vocalization pitch) and the congruency between the response and the movement direction of the stimulus. *Note*. Error bars depict the standard error of the mean. Asterisks indicate statistically significant differences (***p < .001).

Each participant sat in a dimly lit room with his or her head 75 cm in front of a 19″ CRT monitor (screen refresh rate: 85 Hz; screen resolution: 1,280 × 1,024). A head-mounted microphone was adjusted close to the participant’s mouth. At the beginning of each trial, a blank white screen was presented for 2,700 ms. Then the reference stimulus (Figure 3: start_1 or start_2 in Figure 3) was presented for 1,000 ms. After that, the target stimulus (Figure 3: forward_1 or backward_1 when the reference was start_1; forward_2 or backward_2 when the reference was start_2) was presented for 1,000 ms. The size of the stimulus was 3.4° (measured from the swing seat to the top of the head). Participants were required to pronounce the vowel [æ] in a high or low pitch according to the movement direction of the target stimulus (i.e., whether it moves forward or backward). Although we were not interested in the vowel content of the responses, we had to select a vowel for the response because in this way we made sure that the participants had a clear target utterance, and thus the vowel content of high and low responses would be uniform in all responses of every participant. We did not want to select a rounded vowel for this purpose because vowel roundness can increase variation in F2 values within and between participants (Zsiga, 2013). The vowel [æ] was selected for the study because this vowel does not cause biases between the word “*eteenpäin*” (forward; /ete:npæin/) and “*taaksepäin*” (backward; /tɑ:ksepæin/).

The experiment was divided into two blocks that were separated by a 10-min break. In one block, the participants were required to vocalize high-pitched [æ] if the swing moved forward and low-pitched [æ] if it moved backward. In the other block, the participants were required to vocalize low-pitched [æ] if the swing moved forward and high-pitched [æ] if it moved backward. The beginning of each block included a practice session. This session presented 20 repetitions including the same number of hypothetically congruent and incongruent stimulus–response conditions. The block was not, however, started before the participant demonstrated in the practice session that he or she consistently produced responses according to the instructions. Hence, five participants had to perform each of the practice session twice.

Half of the participants performed first the high-forward block. Both blocks consisted of 15 stimulus conditions in which the face of the swinging girl was pointing to the right and the swing moved forward, 15 stimulus conditions in which the face of the swinging girl was pointing to the right and the swing moved backward, 15 stimulus conditions in which the face of the swinging girl was pointing to the left and the swing moved forward, and 15 stimulus conditions in which the face of the swinging girl was pointing to the left and the swing moved backward. All stimuli were presented in randomized order and on a white background. In total, Experiment 2 consisted of 120 trials (30 repetitions × 2 [block] × 2 [pitch]).

The participants were instructed to pronounce the vowel as quickly as possible after the onset of the target stimulus. It was emphasized that the vowel should be produced as a short [æ] rather than a long [æ:] in a natural talking voice. The participants were instructed that the difference between the high- and low-pitched vocalization is adequate if another individual would be able to hear the difference. The experimenter presented example utterances of adequately high and low pitches. During the practice session, the experimenter verified that the vocalizations met these criteria. After the experiment, the participants were asked whether the task was easier to perform in the high-forward/low-backward or in the high-backward/low-forward block.

Sound recording and stimulus presentation were carried out with Presentation® software. The vocal responses were recorded for 2,000 ms starting from the onset of the target object. At the beginning of the experiment, the recording levels were calibrated for each participant using the voice calibration function of Presentation® software (Version 16.1, www.neurobs.com) so that the recording levels would match the natural intensity of the participant’s voice.

#### 3.1.3 Statistical analyses

For analysis purposes, the onsets of the vocalizations were located for each trial as the first observable peak in the acoustic signal by Praat (Boersma, 2001). Similarly, the offsets of the vocalization were located individually for each trial as the observable ending of the acoustic signal. For this task, onsets and offsets were initially located by a highly experienced person who was blind to the condition of each acoustic signal. The spectral components (F1 and F2), as well as F0, were calculated as median values of the middle third of the voiced section of the vowel. The intensity (dB) was calculated as the maximum value of the voiced section. The procedure of locating the onsets and offsets of vocalizations, as well as calculating the acoustic parameters, was carried out in the same way as in our previous studies that similarly investigate reaction times of vocalizations (L. Vainio, 2021).

The following parameters were analyzed from the raw data: reaction times, intensity, F0, F1, and F2. On a few occasions, the formant value was not found by Praat (Version 6.2.15; Boersma, 2001) or the output value clearly exceeded variations that can normally be observed within the voice characteristics of the given vowels (e.g., octave jump errors). The criteria for removing outliers (F1: 400 < æ < 1,100 Hz; F2: 900 < æ < 2,000) was based on the investigation concerning the formants F1 and F2 of the Finnish [æ] (Palo et al., 2012). The criteria were relatively loose because we did not have research-based knowledge about what extent gender and the task (i.e., producing the vowel in a high and low pitch) influence these values. The particularly large number of missing values of F1 is likely to be due to breathy voice quality leading to erroneous estimates some participants produced vocalizations rather quietly, and/or the task of producing high- and low-pitched vowels somehow contaminated the quality of F1 information. Such values, as well as values that were more or less than two standard deviations from a participant’s median (Experiment 1: F0: 1.5%; F1: 37.4%; F2: 3.8%), were discarded prior to analyzing the acoustic characteristics of the vocalizations. Due to the considerably large number of F1 outliers, the outcomes of F1 analysis should be interpreted with caution.

However, prior to analyzing reaction times and any of these vocal parameters, the errors (i.e., the participant uttered the wrong speech unit [3.4% from which 82.2% were performed in incongruent conditions], and the conditions in which a participant did not produce any response [0.8% from which 84.6% were performed in incongruent conditions]) were removed from the data. In the analysis of reaction times, the 4.9% of trials in which the reaction times were more/less than two standard deviations from a participant’s overall mean were removed from the data. In addition, for analyzing fundamental frequencies, the raw F0 values were converted to semitones (st) relative to each participant’s mean F0. Semitone conversion was conducted to account for the logarithmic nature of the perceiving pitch and pitch movements and to eliminate the bimodal distribution of fundamental frequencies caused by the fact that male speakers have fundamentally lower F0 values than female speakers.

The statistical significance of observed differences was tested using the generalized linear mixed model (GLMM) analysis framework. The model that was fitted to the data to predict outcome *i* for participant *j* was common to all the dependent variables (RT, intensity, F0, F1, or F2) and is presented in Equation (1) as follows

(1) $$\begin{array}{l} Y_{i,j}=\beta_{0}+\gamma_{0,j}+(\beta_{1}+\gamma_{1,j})\times C_{i,j}+(\beta_{2}+\gamma_{2,j})\times D_{i,j}+\beta_{3}\times R_{i,j}+\beta_{4}\times (C_{,i,j}\times R_{,i,j})\\ \;\;\;\;\;\;\;\;\;\;+\beta_{5}\times (C_{,i,j}\times D_{,i,j})+\beta_{6}\times (R_{,i,j}\times D_{,i,j})+\beta_{7}\times (C_{,i,j}\times R_{,i,j}\times D_{,i,j})+\varepsilon_{i,j} \end{array}$$

In this equation, *C* = Congruency, *D* = Direction, *R* = response, *β* = a fixed effect coefficient, and *γ* = random effect coefficient. In other words, the GLMM analyses treated Congruency (congruent vs. incongruent), Swinging direction (left vs. right), and Response (high-pitched vs. low-pitched) as fixed within factors. In addition, there was a random intercept of participant with a random slope of Congruency and Swinging direction. Residual distributions differed considerably between the dependent variables of the study, requiring minor adjustments to the model. First, for RT, intensity, and F1, the residual distributions were significantly positively skewed, so a gamma distribution assumption (log link function) was implemented. Second, for F2, the residual distribution was negatively skewed. Therefore, to use the same GLMM as for RT, intensity, and F1 (gamma distribution with log link), we transformed the F2 values so that their distribution was positively skewed. Each F2 value was subtracted from the highest value and as a result, the distribution was flipped with no effect on its shape (nor group means, group variances, and so on). The estimated means were then back-transformed. Finally, for the F0 values converted to semitones, the residual distribution was approximately normal, and normal distribution assumption (identity link function) was implemented. All pairwise comparisons were carried out using Bonferroni correction for multiple comparisons. The analyses were carried out using the SPSS statistics software package (version 28).

### 3.2 Results

The reaction time analysis revealed a significant main effect of Congruency, *F*[1, 2392] = 43.05, *p* < .001. Congruent responses were performed faster (*M* = 483 ms) than incongruent responses (*M* = 602 ms, *d_z_* = 0.99). This effect is presented in Figure 3. In addition, the two-way interaction between Congruency and Swinging direction was significant, *F*[1, 2392] = 5.69, *p* = .017. The congruency effect was slightly larger for the stimuli of right-directed swinging (left direction-congruent: *M* = 486 ms vs. incongruent: *M* = 595 ms; right direction-congruent: *M* = 481 ms vs. incongruent: M = 610 ms). Finally, we analyzed the congruency effect observed reaction times so that Gender was added as an independent variable. This analysis did not reveal a significant interaction between Congruency and Gender (*p* = .126) suggesting that the congruency effect can be observed for both genders.

Regarding voice characteristics, the analysis of intensity revealed a significant main effect of Response, *F*[1, 2516] = 462.59, *p* < .001; high-pitched vocalization: *M* = 78.2 dB; low-pitched vocalization: *M* = 76.9 dB, *d_z_* = 0.23. The analysis of F0 revealed a significant and expected main effect of response, *F*[1, 2478] = 13156.03, *p* < .001; high-pitched vocalization: *M* = 2.4 st; low-pitched vocalization: *M* = –2.9, *d_z_* = 0.95. The interaction between Congruency and Response was also significant, *F*[1, 2478] = 7.10, *p* = .008; high-pitched vocalization: Congruent: *M* = 2.3 st, Incongruent: *M* = 2.5 st, *d_z_* = 0.12; low-pitched vocalization: Congruent: *M* = –2.9 st, Incongruent: *M* = –3.0 st. The analysis of F1 revealed a significant main effect of response, *F*[1, 1572] = 30.62, *p* < .001; high-pitched vocalization: *M* = 591 Hz; low-pitched vocalization: *M* = 567 Hz, *d_z_* = 0.19. In addition, the analysis of F2 revealed a significant main effect of Congruency, *F*[1, 2420] = 13.22, *p* < .001; congruent: *M* = 1,565 Hz; incongruent: *M* = 1,614 Hz, *d_z_* = 0.23 and Response, *F*[1, 2420] = 97.01, *p* < .001; high-pitched vocalization: *M* = 1,620 Hz; low-pitched vocalization: M = 1,560 Hz, *d_z_* = 0.28. The interaction between Congruency and Response was also significant, *F*[1, 2420] = 7.55, *p* = .006; high-pitched vocalization: Congruent: *M* = 1,603 Hz, Incongruent: *M* = 1,636 Hz, *d_z_* = 0.16; low-pitched vocalization: Congruent: *M* = 1,528 Hz, Incongruent: *M* = 1,592 Hz, *d_z_* = 0.30. Finally, 21 (95.5%) participants judged that the task was easier to perform in the high-forward/low-backward block.

### 3.3 Discussion

The results of Experiment 2 show that vocal responses are produced faster in the congruent (high-forward, low-backward) than in the incongruent conditions. This suggests that the effect observed in Experiment 1 can be based on pitch-related features of the auditory stimuli. Importantly, this supports the view that high-pitched vocalizations are implicitly associated with forward-directed movement, and low-pitched vocalizations are associated with backward-directed movement. Regarding the vocal characteristics, intensity, F1 and F2 values were significantly higher for high-pitched responses than for low-pitched responses suggesting that the production of high-pitched vocalization is linked to the increased intensity, the front position of the tongue, as well as the oral cavity. Furthermore, the F0 values of high-pitched vocalization were slightly smaller in the incongruent than congruent condition suggesting a tendency to increase the vocalization pitch when the swing moves backward. However, the effect size of this effect was very small (*d_z_* = 0.12). Regarding F2 values, the significant interaction between Congruency and Response suggested these values are increased when producing high-pitched vocalizations and the swing moves backward, and when producing low-pitched vocalizations and the swing moves forward. This is somewhat inconsistent finding in relation to the finding observed in reaction times, that high-pitched vocalizations are associated with forward-directed movement, whereas low-pitched vocalizations are associated with backward-directed movement. It is possible, for example, that the increased tongue backing in the incongruent conditions reflects increased articulatory effort in these conditions, or some kind of compensatory processes of articulation.

It is possible that the effect observed in Experiments 1 and 2 is a non-generalizable phenomenon that can be only observed in the Finnish culture and in relation to the front–back movement of swinging. Hence, Experiment 3 explores whether the sound symbolism phenomenon observed in Experiment 2 can be generalized to the concepts of front and back. For that purpose, our participants were required to perform the same responses as in Experiments 2. However, as opposed to Experiment 2, in Experiment 3, the participants were performing these responses based on whether the target (a ball) was located at the front of or back of the referent object (a chair).

## 4 Experiment 3

### 4.1 Method

#### 4.1.1 Participants

Twenty-two volunteers naïve to the purposes of the experiment participated in Experiment 3 (21–37 years of age; mean age = 23.2 years; six males; one left-handed). All participants were native speakers of Finnish and reported normal hearing and normal or corrected-to-normal vision. Written informed consent was obtained from all participants. The study was conducted according to the principles expressed in the Declaration of Helsinki. The study was approved by the Ethical Review Board in the Humanities and Social and Behavioral Sciences at the University of Helsinki.

#### 4.1.2 Stimuli, procedure, and apparatus

**Figure 4.**
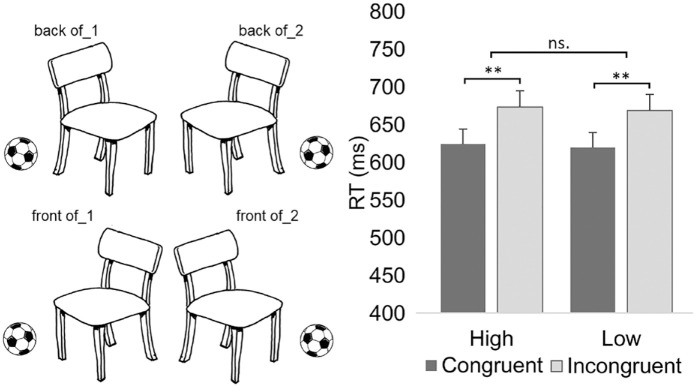
(Left)The stimuli used in Experiment 3. (Right) The mean vocal reaction times for Experiment 3 as a function of the response (vocalization pitch) and the congruency between the response and the front/back position of the target object (a ball) in relation to the reference object (a chair). *Note*. Error bars depict the standard error of the mean. Asterisks indicate statistically significant differences (**p < .010).

The apparatus, environmental conditions, and calibration were the same as those in Experiment 2. The procedure and design were similar to that of Experiment 2 with the exception that vocal responses were performed based on whether the target ball was located at the front of or back of the chair (see stimuli in Figure 4). The chair was pointing to the right or left. The chair was always situated at the same location at the center of the screen regardless of its pointing direction. The ball was located on the right or left side of the chair. The ball was situated at the same location regardless of whether it was at the front or back of the chair when the ball was located on the left and right side of the chair. The size of the stimuli, including the chair and the ball, was 8.4° (horizontally and vertically). Similar to Experiment 2, the vowel [æ] was selected for the study because this sound does not cause biases between the word “*edessä*” (front of) and “*takana*” (back of). That is, if any, the vowel [æ] should produce response facilitation in incongruent conditions due to associating the vowel with the front position of the ball.

Similar to Experiment 2, Experiment 3 was divided into two blocks that were separated by a 10-min break. At the beginning of each trial, a blank white screen was presented for 2,700 ms. Then the target stimulus (Figure 4: front of_1, front of_2, back of_1, or back of_2) was presented for 1,500 ms. All stimuli were presented in randomized order and on a white background. The vocal responses were recorded for 2,000 ms starting from the onset of the target object. The participants were instructed to respond in the same way as in Experiment 2. That is, in Experiment 3, they were asked to produce either a high or low-pitched [æ] based on whether the ball was at the front or back of the chair.

The beginning of each block included a practice session that presented 20 repetitions including the same number of hypothetically congruent and incongruent stimulus-response conditions. In total, Experiment 3 consisted of 120 trials (30 repetitions × 2 [block] × 2 [pitch]). After Experiment 3, the participants were asked whether the task was easier to perform in the high-front/low-back or in the high-back/low-front block.

#### 4.1.3 Statistical analyses

The data were processed in the same way as in Experiment 2. The following parameters were analyzed from the raw data: reaction times, dB, F0, F1, and F2. Regarding the voice characteristics, the same criteria were employed for removing outliers (F0: 1.6%;F1: 40.5%; F2: 8.5%) as in Experiment 2. Due to the considerably large number of F1 outliers, the outcomes of F1 analysis should be interpreted with caution. Prior to analyzing any of these parameters, the errors (i.e., the participant uttered the wrong speech unit [1.5% from which 82.5% were performed in incongruent conditions], and the conditions in which a participant did not produce any response [0.9% from which 45.8% were performed in incongruent conditions]) were removed from the data. In the analysis of reaction times, 4.4% of trials in which the reaction times were more/less than two standard deviations from a participant’s overall mean were removed from the data.

The statistical significance of observed differences was tested using the GLMM analysis framework. The model that was fitted to the data to predict outcome *i* for participant *j* was common to all the dependent variables (RT, intensity, F0, F1, or F2) and is presented in Equation (2) as follows

(2) $$Y_{i,j}=\beta_{0}+\gamma_{0,j}+(\beta_{1}+\gamma_{1,j})\times C_{i,j}+\beta_{2}\times R_{i,j}+\beta_{3}\times (C_{,i,j}\times R_{,i,j})+\varepsilon_{i,j}$$

In the equation, *C* = Congruency, *R* = Response, *β* = a fixed effect coefficient, and *γ* = random effect coefficient. In other words, the GLMM analyses treated Congruency (congruent vs. incongruent) and Response (high-pitched vs. low-pitched) as fixed within factors. In addition, there was a random intercept of participant with a random slope of Congruency. Residual distributions for the different dependent variables were as in Experiment 2, and the same modifications to the GLMMs were thus applied. All pairwise comparisons were carried out using Bonferroni correction for multiple comparisons. The analyses were carried out using the SPSS statistics software package (version 28).

### 4.2 Results

The analysis of reaction times revealed the significant main effect of Congruency, *F*[1, 2458] = 9.66, *p* = .002. Congruent responses were performed faster (*M* = 621 ms) than incongruent responses (*M* = 671 ms, *d_z_* = 0.37). This effect is presented in Figure 4. We also analyzed the congruency effect observed reaction times so that Gender was added as an independent variable. This analysis did not reveal a significant interaction between Congruency and Gender (*p* = .335) suggesting that the congruency effect can be observed for both genders. Moreover, because the swinging direction was observed to produce an interaction effect between Congruency and Direction in Experiment 2, we analyzed reaction times so that the target side was added as an independent variable to the model in addition to Congruency and Response. However, given that this direction information was not observed to influence vocal characteristics in Experiment 2, we investigated how the target side influences responses only in relation to reaction times. The target side was not observed to influence reaction times (the main effect of target side: *p* = .879; Congruency × Side: *p* = .551; Response × Side: *p* = .960; Congruency × Response × Side: *p* = .063).

Regarding voice characteristics, the analysis of intensity revealed a significant main effect of Response, *F*[1, 2572] = 274.27, *p* < .001; high-pitched vocalization: *M* = 79.3 dB; low-pitched vocalization: *M* = 78.3 dB, *d_z_* = 0.26. The analysis of F0 also revealed a significant and expected main effect of Response, *F*[1, 2530] = 13,559.89, *p* < .001; high-pitched vocalization: *M* = 2.7 st; low-pitched vocalization: *M* = –3.5 st, *d_z_* = 0.97, as well as the interaction between Congruency and Response, *F*[1, 2530] = 8.45, *p* = .004; high-pitched vocalization: Congruent: *M* = 2.9 st, Incongruent: *M* = 2.5 st, *d_z_* = 0.19; low-pitched vocalization: Congruent: *M* = –3.5 st, Incongruent: *M* = –3.6 st. The analysis of F1 revealed a significant main effect of Response, *F*[1, 1528] = 145.95, *p* < .001; high-pitched vocalization: *M* = 600 Hz; low-pitched vocalization: *M* = 546 Hz, *d_z_* = 0.41. In addition, the analysis of F2 revealed a significant main effect of Response, *F*[1, 2353] = 73.10, *p* < .001; high-pitched vocalization: *M* = 1,630 Hz; low-pitched vocalization: *M* = 1,578 Hz, *d_z_* = 0.22. Finally, 17 (80.9%) participants judged that the task was easier to perform in the high-front/low-back block.

### 4.3 Discussion

The results of Experiment 3 show that vocal responses are produced faster in the congruent (high-front, low-back) than in the incongruent conditions. This suggests that the finding observed in Experiment 2 does not present an isolated effect associating high- and low-pitched vocalizations solely with a particular movement direction, but rather it seems that this sound-space symbolism effect operates within more generalizable conceptualization processes in which particular vocalizations are systematically associated with the concepts of forward/front and backward/back. Again, the analysis of the vocal characteristics showed that intensity, F1, and F2 values were significantly higher for high-pitched responses than for low-pitched responses. This can be taken to suggest that high-pitched vocalizations, in comparison with low-pitched vocalizations, are produced with increased intensity, the oral cavity and the front position of the tongue. Finally, the analysis of F0 values showed that these values are slightly higher for the congruent than incongruent responses. This might suggest that vocalizations are produced even higher when the target is in the front of the chair. However, given that in Experiment 2 the effect was opposite to that observed in Experiment 3, and given that the effect sizes of these effects are very small (Experiment 2: *d_z_* = 0.12; Experiment 3: *d_z_* = 0.19), it would not be justifiable to discuss these effects further.

## 5 General discussion

This study showed that high-pitched vocalizations are associated with the concepts of forward and front, whereas low-pitched vocalizations are associated with the concepts of backward and back. Previous observations have presented corresponding interactions between the high- and low-pitched sound and, for example, visual elevation (the pitch-elevation effect: Ben-Artzi & Marks, 1995; Bernstein & Edelstein, 1971) and size (Gallace & Spence, 2006) associating high-pitch sounds with smaller visual sizes and an upper visual position, whereas low-pitch sounds are associated with larger visual sizes and a lower visual position. However, in comparison with those kinds of cross-modal correspondence phenomena, this study presented similar interaction between the speech sounds and the meaning of spatial concepts. Hence, the study can be taken to demonstrate a novel sound-space symbolism effect instead of an audiovisual correspondence phenomenon.

The theoretically interesting and critical question is why high/low vocalizations are implicitly associated with these spatial concepts. One possibility is that the effect observed in this study has the same basis as the pitch-elevation effect. It is possible that there is some semantic overlap between the concepts up and front as well as down and back (Koch et al., 2011). If this were the case, one might assume that in addition to linking high-pitched sounds to the concept of up and low-pitched sounds to the concept of down, the high- and low-pitched sounds could be correspondingly linked to the concepts of front and back, respectively. It has been shown that one explanation for the pitch-elevation effect might be that higher frequencies relatively frequently originate from elevated sources in the natural environment (Parise et al., 2014). However, it is rather implausible that higher frequencies would originate more frequently from (egocentrically) the front of a person than the back of a person making this explanation for the present effect improbable. As such, it is difficult to form any explanation for the present effect, which assumes that the effect ultimately originates from such environmentally based acoustic correspondences between high- and low-pitched sounds and the spatial dimensions of front and back.

One possibility to explain the effect is not necessarily associated with the F0 of high- and low-pitched vocalizations per se, but rather some articulatory properties that are linked to producing high- and low-pitched vocalizations. Therefore, the results of the analysis carried out for vocal characteristics could reveal something from the mechanisms behind the effect. First, the high-pitched vocalizations were associated with increased vocalization intensity in comparison with the low-pitched vocalization in Experiments 2 and 3. However, the effect was observed in Experiment 1 even though the intensity was identical in the high- and low-pitched stimuli. Perhaps this allows us to rule out the possibility that the effect is based on variations in vocalization intensity. The more likely explanation for the effect emphasizes that the high-pitched vocalizations were associated with the heightened F1 and F2 in comparison with the low-pitched vocalization in Experiments 2 and 3. This, in turn, suggests that the oral cavity (linked to heightened F1 values) and the front position of the tongue (linked to heightened F2 values) were increased in relation to the high-pitched vocalizations. It has been proposed that the lifting/lowering of the larynx (Honda et al., 1999), different forward/backward movements of the tongue, and mechanical coupling between the tongue and the larynx can be, to some extent, associated with controlling the intended pitch (Ladefoged, 1968). This view holds that tongue protrusion can be involved in producing high-pitched vocalizations because it benefits the anterior pull of the hyoid bone and the thyroid cartilage leading to increased stiffness of the vocal folds (S. Sapir, 1989). Indeed, the previous research (Higashikawa et al., 1996; Saldías et al., 2021) suggests that when producing a high-pitched voice, frequencies of F1 and F2 can be increased perhaps due to lifting of the larynx as well as increasing the pharyngeal cavity and the front position of the tongue. In contrast, when producing a low-pitched voice, the larynx is lowered, the back position of the tongue is increased, and the pharyngeal cavity is relatively decreased resulting in relatively decreased frequencies of F1 and F2. Importantly, this is what was also observed in our study. A somewhat corresponding phenomenon can be observed in the phenomenon of intrinsic vowel pitch as discussed in the Introduction (Whalen & Levitt, 1995).

As such, the current finding can be based on the articulatory features of high and low-pitched vocalizations that manifest themselves in the modulations of F1 and F2. It has been proposed that associating round vowels with round shapes is based on the articulatory shape, and associating open vowels with large sizes is based on the increased oral cavity (Ramachandran & Hubbard, 2001). Similarly we propose that one possible candidate for explaining the effect observed in this study is linked to the front/back position of the tongue together with increasing the pharyngeal cavity and lifting/lowering of the larynx, that according to F1 and F2 values seems to vary between high- and low-pitched vocalizations. In particular, we propose that the sound-space symbolism effect observed in this study is based, to large extent, on grounding the conceptual representations of forward/front and backward/back in articulatory motor processes that move the tongue forward and backward. This perspective is supported by our recent study, which demonstrated that the production of a front vowel is associated with the concepts of forward and front, whereas the production of a back vowel is associated with the concepts of backward and back (L. Vainio et al., 2023).

At this point, it has to be emphasized that there was an equal chance that the required response was either high [æ] or low [æ] in the upcoming trial of Experiments 2 and 3. Due to this, it was most likely that to achieve the optimal performance, the tongue was implicitly shaped—between the experimental trials—to a speech-ready position, which was adapted to the position optimally ready to execute both of the speech sounds in the block (cf. Šimko & Cummins, 2010). Something similar occurs normally in speech, in the phenomenon called anticipatory co-articulation, in which the shape of the vocal tract at a given time is a compromise between the previous and the potentially upcoming articulatory gesture (Hardcastle & Hewlett, 2006). Hence, we have a reason to assume that the task indeed involved a slight front/back-directed tongue movement into the front/back position to perform the high/low-pitched vocalizations, as explained above. As a consequence, we propose that producing high/low-pitched vocalizations mechanically involves moving the tongue to the front/back positions, which might link these speech sounds to the concepts of front and back, respectively. However, this account warrants further investigation.

Why would then the spatial concepts of front/forward and back/backward be rooted in articulatory motor representations? Our account is largely based on the theory of embodied cognition according to which concepts are grounded in the sensorimotor systems (Barsalou, 2008). Although this is assumed to be the case, particularly in relation to concrete concepts (e.g., the concept of grasping is grounded in the hand-motor area), it has been proposed that motor processes are also involved in representing relatively abstract concepts such as opening/closing (Glenberg & Kaschak, 2002) that might be represented in relation to the corresponding hand and mouth movements (Borghi et al., 2018; L. Vainio, 2019). Therefore, given that the concepts of forward and backward are associated with forward- and backward-directed limb movements (L. Vainio et al., 2015, 2019), it is possible that the representations of these concepts are also grounded in forward- and backward-directed articulatory tongue movements.

Now let us come back to the anecdotal assumption, stated at the beginning of the paper, that people might show a tendency to utter high-pitch voices in relation to forward-directed swing movement and low-pitched voices in relation to backward-directed swing movement as if they were imitating the front-and-back swinging movement with their vocal apparatus. Based on this study, this indeed seems to be the case, although it should be emphasized that we do not know yet whether this phenomenon is exclusively associated with the Finnish or whether it is a more universal phenomenon. The account that might be adapted to theoretically explain this observed phenomenon is the so-called mouth-gesture hypothesis (Paget, 1944; Wallace, 1881). The hypothesis assumes that people have a tendency to roughly and implicitly imitate manual signs or their external referents using lips and tongue. It emphasizes that the goal of this imitation is not solely to produce sounds that bear any acoustic resemblance to these external events, but rather to produce mouth gestures that accompany these events. With a slight elaboration, this hypothesis could be assumed to also explain the phenomenon observed in this study. According to this view, people prefer to produce the high-pitched /ki:/ and the low-pitched /kɑ:/ with the front-and-back swinging movement, respectively, because of this innate proclivity to copy external events, such as rhythmic and repetitive movements, with the movements of their articulators; That is, to move their tongue forward (notice that [i] is a front vowel, and that high-pitched vocalizations seem to be produced by a relatively front position of the tongue) and backward in imitative synchrony with the front-and-back movement of the swing.

### 5.1 The limitations of the study

The study had some limitations that warrant further investigation. Experiment 3 was conducted to show that the effect is generalizable instead of solely linked to swinging movement. Nevertheless, one might still argue that the effect is based on a culture-specific phenomenon, observed specifically in the Finnish language environment so that people have learned to associate raising pitch with the forward-directed swing movement and lowering pitch with the backward-directed swing movement. This assumption holds that this association might be abstracted to influence even representing the concepts of front of and back of, which in turn can be observed in the outcome of Experiment 3. However, it should be notified that none of the participants were explicitly aware that the forward-directed swing movement is typically associated with the raising pitch and the backward-directed swing movement is associated with the lowering pitch. Although the participants might have also learned this association implicitly, the question of why they show this association in the first place remains valid. The other reason why this phenomenon should be investigated in various language environments and in a wider range of contexts is that the Finnish words for forward/front include the front vowel [e] (with higher intrinsic F0), whereas the words for backward/back include the back vowel [ɑ] (with lower intrinsic F0). This fact might provide a language-specific ground for observing the effect. Finally, it is possible that the intonational contours of the high- and low-pitched vocalizations might have contributed to the effect observed in the study. Indeed, evidence suggests that prosody is an important instrument for iconicity (Dingemanse et al., 2016; L. Vainio & Vainio, 2021). Although we failed to reliably detect any intonational contours in our vocalization data, perhaps because short vowels in the Finnish are typically tonally static (M. Vainio et al., 2010; the fact that the vocalizations were approximately 160 ms long underlines this issue), this aspect should be addressed in the future studies.

In conclusion, this study showed that high-pitched vocalizations are associated with the concepts of front/forward, whereas low-pitched vocalizations are associated with the concepts of back/backward. Furthermore, based on the analysis of vocal characteristics, we proposed that this effect is linked to a specific manner of articulation required for vocalizing high- and low-pitched utterances. High-pitched utterances are produced by a relatively front position of the tongue, together with the larynx lifting and increasing the pharyngeal cavity, associating them with the concepts of forward and front. In contrast, low-pitched utterances are produced by a relatively back position of the tongue, together with the larynx lowering and decreasing the pharyngeal cavity, associating them with the concepts of backward and back. In addition to presenting a novel sound-space symbolism effect, these observations contribute to an understanding of mechanisms in sound-space linkages and more generally in sound symbolism.
